# A Novel Single-Cell FISH-Flow Assay Identifies Effector Memory CD4^+^ T cells as a Major Niche for HIV-1 Transcription in HIV-Infected Patients

**DOI:** 10.1128/mBio.00876-17

**Published:** 2017-07-11

**Authors:** Judith Grau-Expósito, Carla Serra-Peinado, Lucia Miguel, Jordi Navarro, Adrià Curran, Joaquin Burgos, Imma Ocaña, Esteban Ribera, Ariadna Torrella, Bibiana Planas, Rosa Badía, Josep Castellví, Vicenç Falcó, Manuel Crespo, Maria J. Buzon

**Affiliations:** a Department of Infectious Diseases, Hospital Universitari Vall d’Hebrón, Institut de Recerca (VHIR), Universitat Autònoma de Barcelona, Barcelona, Spain; b Department of Pathology, Hospital Vall d’Hebron, Universitat Autònoma de Barcelona, Barcelona, Spain; c Unit of Infectious Diseases, Complexo Hospitalario Universitario de Vigo, IIS Galicia Sur, Vigo, Spain; Albert Einstein College of Medicine

**Keywords:** viral persistence, viral reactivation, viral reservoirs, human immunodeficiency virus

## Abstract

Cells that actively transcribe HIV-1 have been defined as the “active viral reservoir” in HIV-infected individuals. However, important technical limitations have precluded the characterization of this specific viral reservoir during both treated and untreated HIV-1 infections. Here, we used a novel single-cell RNA fluorescence *in situ* hybridization-flow cytometry (FISH-flow) assay that requires only 15 million unfractionated peripheral blood mononuclear cells (PBMCs) to characterize the specific cell subpopulations that transcribe HIV RNA in different subsets of CD4^+^ T cells. In samples from treated and untreated HIV-infected patients, effector memory CD4^+^ T cells were the main cell population supporting HIV RNA transcription. The number of cells expressing HIV correlated with the plasma viral load, intracellular HIV RNA, and proviral DNA quantified by conventional methods and inversely correlated with the CD4^+^ T cell count and the CD4/CD8 ratio. We also found that after *ex vivo* infection of unstimulated PBMCs, HIV-infected T cells upregulated the expression of CD32. In addition, this new methodology detected increased numbers of primary cells expressing viral transcripts and proteins after *ex vivo* viral reactivation with latency reversal agents. This RNA FISH-flow technique allows the identification of the specific cell subpopulations that support viral transcription in HIV-1-infected individuals and has the potential to provide important information on the mechanisms of viral pathogenesis, HIV persistence, and viral reactivation.

## IMPORTANCE

Persons infected with HIV-1 contain several cellular viral reservoirs that preclude the complete eradication of the viral infection. Using a novel methodology, we identified effector memory CD4^+^ T cells, immune cells preferentially located in inflamed tissues with potent activity against pathogens, as the main cells encompassing the transcriptionally active HIV-1 reservoir in patients on antiretroviral therapy. Importantly, the identification of such cells provides us with an important target for new therapies designed to target the hidden virus and thus to eliminate the virus from the human body. In addition, because of its ability to identify cells forming part of the viral reservoir, the assay used in this study represents an important new tool in the field of HIV pathogenesis and viral persistence.

## INTRODUCTION

HIV-1 infects mainly CD4^+^ T cells and is able to establish viral latency extremely soon after the initial viral infection (1, 2). Administration of antiretroviral therapy (ART) efficiently decreases the plasma viral load in the plasma of HIV-infected patients but does not fully eliminate HIV-1 (3). The presence of latently infected cells that are able to reinitiate new rounds of viral replication after ART withdrawal (4–6) and the existence of low-level ongoing viral replication (7), most likely in tissue reservoirs (8–10), represent the main obstacles to the complete eradication of HIV-1 from the human body.

The identification of a biomarker that exclusively identifies latently infected cells in HIV-infected patients remains a challenge. Truly latently infected cells have an integrated copy of HIV inserted within the cell genome and do not produce viral RNA transcripts and proteins; thus, infected cells harboring latent HIV can barely be distinguished from their uninfected counterparts. A new study has recently identified a potential cell marker for latently infected cells; CD32a was found to be upregulated in the latent portion of HIV-infected cells in approximately 50% of all reservoir cells (11).

The proportion of latently infected cells able to produce replication-competent virus is very small; conservative measures using the gold standard quantitative viral outgrowth assay (qVOA) estimate that the fraction of this latent reservoir is about one infected cell in a million resting CD4^+^ T cells in ART-treated individuals (12, 13). The characterization of this specific viral reservoir has been hampered for years because of the extremely low percentage of latently infected cells. Detection of viral nucleic acids by PCR-based methods is an alternative approximation to identify HIV reservoir cells (14). Quantification of viral DNA is being used in current cure-related clinical trials as a marker of therapy effectiveness (15, 16). The total HIV DNA value correlates well with the qVOA assay and the integrated forms of HIV (17–19). In addition, the total HIV-1 DNA inversely correlates with the time to viral rebound after therapy interruption in patients who are treated early after infection (20, 21). Viral DNA quantification has been used to identify the cell composition of the HIV reservoir after the specific isolation of populations of interest; for instance, central memory, transitional memory, effector memory, stem cell memory, and follicular CD4^+^ T cells have been identified as the main cell subsets supporting HIV persistence in patients on ART (22–25). However, a significant disadvantage of using HIV DNA as a surrogate marker of viral reservoir cells is that most of the viral DNA is defective and consequently will never produce fully replicative viral particles (13). Another significant disadvantage of using HIV DNA detection to study the cell composition of the HIV reservoir is the requirement of previous cell isolation, making difficult the identification of reservoir cell subsets that represent a small portion of the total pool of infected cells.

Cells expressing HIV RNA encompass a subset of the total viral reservoir that contains cells actively transcribing HIV. This specific reservoir has been defined by Pasternak et al. as the “active viral reservoir” (26). Importantly, a fraction of resting CD4^+^ T cells are capable of expressing HIV RNA without the concomitant production of viral particles in both ART-treated and untreated HIV-infected patients (27–29). Cell-associated HIV RNA quantification strongly correlated with disease progression and inversely correlated with CD4^+^ T cell counts in untreated viremic patients (30–33). Indeed, unlike plasma viremia, intracellular HIV RNA levels significantly increase over time during untreated infection (31), and levels of intracellular HIV RNA have been associated with virological failure in patients on ART (26). The identification of such infected cells in both treated and untreated HIV-infected persons will provide important information regarding the characterization of cells with transcriptionally active HIV, redirecting the current efforts in the design of new therapies targeting this active viral reservoir. In addition, as HIV infection frequencies and HIV transcription levels might differ by cell type and anatomic location (34), the identification of such infected cells will allow us to determine the specific contribution of HIV to the cellular processes and functions of this active viral reservoir at different anatomical sites and possible cellular sources of residual plasma viremia and the release of viral particles after viral reactivation.

In this study, we have validated a novel RNA fluorescence *in situ* hybridization-flow cytometry (FISH-flow) technique that detects intracellular HIV RNA molecules at the single-cell level in 15 million primary unfractionated peripheral blood mononuclear cells (PBMCs) from HIV-infected individuals. Using this novel assay, we have characterized the cells expressing HIV RNA after *ex vivo* HIV infection of unstimulated PBMCs, in primary PBMC samples from ART-treated and untreated HIV-infected patients, and after *ex vivo* viral reactivation of primary CD4^+^ T cells. We found that in samples from HIV-infected patients, the proportion of cells carrying viral transcripts correlated very well with plasma viral loads and intracellular levels of HIV RNA measured by conventional methods and inversely correlated with the absolute numbers and percentages of CD4^+^ T cells and CD4/CD8 ratios. The majority of cells supporting HIV transcription had an effector memory CD4^+^ T cell phenotype. Moreover, we observed that after *ex vivo* infection of unstimulated PBMCs, HIV-infected T cells upregulated the expression of the newly identified marker of latently infected cells CD32. In addition, using this novel RNA FISH-flow assay, we detected reactivation of HIV from primary CD4^+^ T cell samples from patients with undetectable plasma viral loads after *ex vivo* exposure to an activating stimulus. This investigation characterized the cellular sources of active viral reservoirs and identified effector memory CD4^+^ T cells as the main subset expressing intracellular HIV RNA in both untreated and treated HIV-infected individuals. In addition, it provides a useful tool to evaluate the effectiveness of different latency reversal agents (LRAs) in different cell subpopulations.

## RESULTS

### Detection of HIV expression and viral protein production after *ex vivo* infection of unstimulated PBMCs.

A high-sensitivity target-specific set of 50 individual probes targeting the HIV RNA Gag-Pol sequence (bases 1165 to 4402 of the HXB2 consensus genome) was used for HIV RNA detection by the RNA FISH-flow method (Human PrimeFlow RNA Assay; eBioscience). We chose the Gag-Pol region of HIV-1 because it detects unspliced forms of viral transcripts. Importantly, cells containing unspliced HIV RNA decay very slowly after ART initiation and positive cells are successfully observed in patients on ART (35, 36).

**FIG 1:**
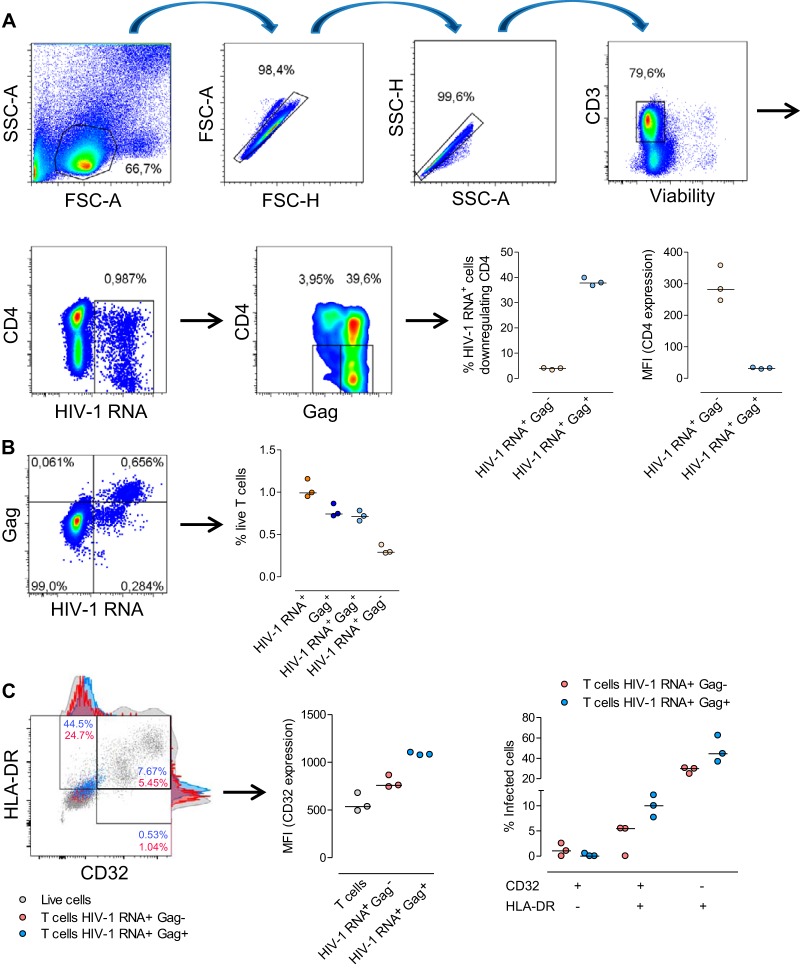
Detection of HIV transcripts and the viral Gag p24 protein in *ex vivo*-infected samples by the RNA FISH-flow assay. Unfractionated and unstimulated PBMCs from healthy donors were infected *ex vivo* with exogenous HIV strain NL4.3. Five days after the initial infection, cells were subjected to the RNA FISH-flow protocol. (A) The graphs on the left are representative flow cytometry plots of HIV transcript detection and CD4 downregulation in Gag p24^+^ T cells. The graphs on the right show the data summary of CD4 downregulation and the MFI (mean fluorescence intensity) of CD4 expression in cells expressing HIV RNA with and without production of the viral Gag p24 protein. (B) Left, representative flow cytometry plot of the dual staining of T cells for HIV RNA and Gag p24 protein. The graph on the right summaries the percentages of infection of the different combinations of cells costained for detection of HIV RNA and the viral Gag p24 protein in three different infected HIV-negative donors. (C) Left, representative flow cytometry plot of CD32 and HLA-DR coexpression in HIV-infected cells. Infected cells expressing only HIV RNA (red) or expressing viral RNA and Gag p24 protein (blue) are overlaid on the whole live-cell population (gray). The graph in the center shows the MFI of CD32 expression in cells expressing only viral RNA, cells expressing HIV RNA and p24, and uninfected T cells. The graph on the right shows the percentages of infected cells expressing CD32 and the HLA-DR markers.

To initially investigate the ability of the new RNA FISH-flow assay to detect HIV expression, unstimulated PBMCs from healthy donors were infected *ex vivo*. We observed consistently robust HIV RNA expression (~1% of all cells) in T cells in all *ex vivo*-infected cells from HIV-negative donors (Fig. 1A and B). Approximately 40% of these HIV RNA-expressing cells concomitantly expressed the viral Gag p24 protein and downregulated the CD4 surface marker, whereas HIV RNA-expressing cells lacking Gag p24 protein production had high CD4 expression. Thus, a majority (~60%) of the HIV-transcribing cells did not downregulate the CD4 cell receptor (Fig. 1A). Using this technique, we were able to distinguish two subpopulations of HIV-expressing cells with high and low RNA transcript levels (Fig. 1B). Cells expressing low levels of HIV transcription were not able to produce detectable viral proteins (~30% of all HIV-expressing cells). Therefore, high production of viral transcripts was needed for concomitant detection of the p24 viral protein and downregulation of the CD4 cell surface marker. Additionally, we investigated the expression of the newly discovered marker of latently infected cells, CD32, by using our system of *ex vivo* infection of unstimulated PBMCs. We observed that HIV-infected T cells expressing viral RNA and the Gag p24 protein upregulated CD32 expression (~2-fold increase), while the increase in the expression of CD32 was less intense in cells expressing only viral RNA (~1.5-fold increase). A slight increase in the proportion of cells expressing CD32 was also observed upon cell infection (~10% of all infected cells). The CD32 expression level, however, was considered low compared to that of non-T cells (Fig. 1C). We also observed the expression of HIV RNA transcripts and viral Gag p24 protein in non-T-cell populations (see Fig. S1A and B in the supplemental material). In contrast to infected T cells, most of the infected non-T cells had simultaneous expression of HIV RNA, Gag p24, and the CD4 receptor (~1%) (Fig. S1B). More phenotypic experiments are certain to further delineate the specific non-T-cell subpopulations supporting HIV replication or viral capture after the *ex vivo* infection of primary cells.

10.1128/mBio.00876-17.1 FIG S1  Detection of HIV RNA and Gag p24 protein in non-T cells. Unfractionated and unstimulated PBMCs from healthy donors were infected *ex vivo* and subjected to the RNA FISH-flow protocol after 5 days of infection. (A) Gating strategy used for the identification of HIV transcription and protein production in non-T cells. (B) Summary data of three HIV-negative donors. Download FIG S1, PDF file, 1.4 MB. Copyright © 2017 Grau et al. 2017 Grau et al. This content is distributed under the terms of the Creative Commons Attribution 4.0 International license.

Next, we investigated the linearity of the assay. Latently infected J-Lat cells with detectable basal HIV expression (clone 9.2) were spiked with the noninfected lymphoid cell line MOLT-4 CCR5+ at different ratios (1/3 dilutions), and the mixture was then subjected to the RNA FISH-flow protocol. HIV RNA expression determined by the experimental curve showed robust consistency with the predicted curve at all of the dilutions tested (down to 50 positive events per million cells) (Fig. S2A). Similar results were observed when the linearity assay was performed with expanded infected cells from HIV-infected individuals (Fig. S2B).

10.1128/mBio.00876-17.2 FIG S2  Linearity of the RNA FISH-flow assay with highly sensitive probes for detection of HIV transcription. (A) The latently infected cell line J-Lat (clone 9.2) spiked with the uninfected MOLT CCR5+ cell line was used to perform the quantification of predicted (blue symbols) versus experimental (orange symbols) results with single expression of HIV RNA. (B) Infection of primary CD4^+^ T cells from HIV-infected patients was expanded *in vitro*, and infected cells were diluted with uninfected cells to perform the quantification of predicted (blue symbols) versus experimental (orange symbols) values of HIV RNA expression measured by the RNA FISH-flow assay. Assay linearity was assessed by linear regression. Download FIG S2, EPS file, 1.1 MB. Copyright © 2017 Grau et al. 2017 Grau et al. This content is distributed under the terms of the Creative Commons Attribution 4.0 International license.

Overall, these experiments demonstrated that with a high-sensitivity set of probes against the Gag-Pol HIV RNA sequence, the RNA FISH-flow assay is a valid method for detection of HIV RNA expression and viral production after the *ex vivo* infection of unstimulated PBMCs.

### Detection of CD4^+^ T cells expressing viral transcripts in samples from HIV-infected patients.

Once the assay was validated with *ex vivo* HIV-infected PBMCs, we determined the percentage of HIV-expressing cells in primary samples from HIV-infected individuals. We observed that samples from HIV-negative donors had a background signal in the HIV RNA detection channel, and this unspecific staining varied between experiments. For this reason, every independent experiment included at least one uninfected donor to normalize the percentage of HIV-expressing cells. Figure S3 shows the raw HIV RNA expression data for additional HIV-infected patients with different plasma viral loads. Normalization of the data was carried out by subtracting the percentage of positive cells in the HIV RNA detection channel of the negative control from the percentage of positive cells measured in each population. After data normalization, the assay was highly reproducible between different experiments (Fig. S4A). To test the specificity and background signal of the highly sensitive set of probes used in this assay, we detected HIV RNA by confocal microscopy in a lymph node tissue sample from an acutely HIV-infected patient (patient 41; Table S1). Figure S4B shows the clear detection of HIV transcripts in tissue from an infected patient. Specifically, we observed two HIV RNA expression patterns, highly positive cells corresponding to HIV RNA-expressing cells and small punctate structures compatible with the capture of HIV virions by follicular dendritic cells as previously documented (37). Of note, HIV RNA staining was not observed in the lymph node of the HIV-uninfected control.

10.1128/mBio.00876-17.3 FIG S3  Representative flow cytometry plots of HIV RNA detection in CD4^+^ T cells from two uninfected (HIV^−^) persons, two ART-treated patients (plasma viral loads of <20 copies/ml), and six untreated patients with different plasma viral loads. Download FIG S3, EPS file, 2.6 MB. Copyright © 2017 Grau et al. 2017 Grau et al. This content is distributed under the terms of the Creative Commons Attribution 4.0 International license.

10.1128/mBio.00876-17.4 FIG S4  Reproducibility of the assay and specificity of the HIV RNA probe. (A) Reproducibility of the assay. Normalized values of HIV RNA expression in CD4^+^ T cells from three different patients assayed in independent experiments (patients 32, 35, and 37; Table S1). (B) HIV RNA-expressing cells imaged by confocal microscopy in a lymph node tissue sample from an uninfected donor (top) and an HIV-infected individual (patient 41, Table S1) (bottom). DAPI staining is blue, and HIV RNA transcripts are red. Clear symbols correspond to values below the limit of detection. Download FIG S4, EPS file, 4.3 MB. Copyright © 2017 Grau et al. 2017 Grau et al. This content is distributed under the terms of the Creative Commons Attribution 4.0 International license.

10.1128/mBio.00876-17.7 TABLE S1  Characteristics of the patients included in this study. Download TABLE S1, DOCX file, 0.02 MB. Copyright © 2017 Grau et al. 2017 Grau et al. This content is distributed under the terms of the Creative Commons Attribution 4.0 International license.

**FIG 2:**
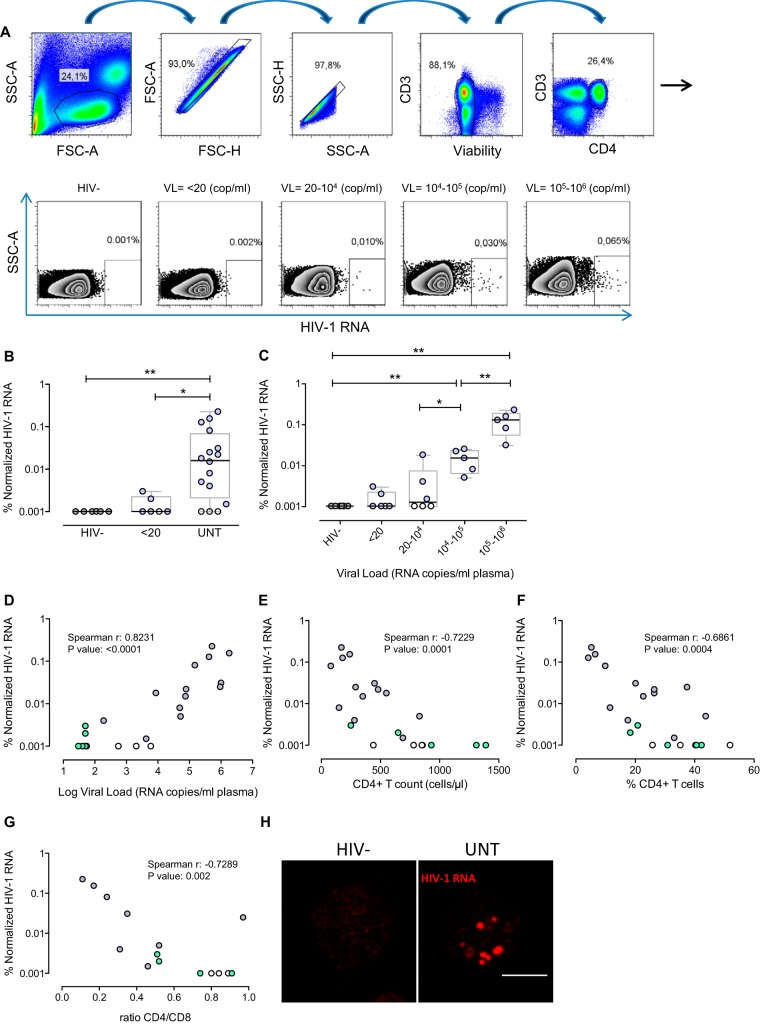
Detection of CD4^+^ T cells expressing HIV RNA transcripts in primary samples from HIV-infected patients. Fifteen million PBMCs from healthy donors and HIV-1-infected patients were thawed and subjected to the RNA FISH-flow protocol for viral transcript detection without any previous cell stimulation. (A) Lymphocytes were gated by using the forward and side scatter areas (FSC and SSC, respectively), and the debris was excluded from the analysis. Cell doublets then were removed from the analysis (FSC-A versus FSC-H, followed by SSC-A versus SSC-H), and live cells were selected by live/dead staining. HIV RNA expression was identified in viable CD4^+^ T cells. Shown are representative flow cytometry plots of HIV RNA detection in CD4^+^ T cells from a uninfected person (HIV^−^), an ART-treated patient (plasma viral load [VL] = <20 copies [cop]/ml;), and untreated (UNT) patients with different plasma VLs. (B) Summary data of normalized HIV RNA^+^ frequency in CD4^+^ T cells in uninfected controls (HIV^−^; *n* = 6), patients treated with ART (plasma VL of <20 copies/ml; *n* = 6), and untreated patients (*n* = 16). (C) Frequency of HIV RNA detection stratified by plasma VLs. (D) Correlation of HIV RNA^+^ cells and plasma VLs. (E) Correlation of percent HIV RNA^+^ cells and absolute numbers of CD4^+^ T cells in blood. (F) Correlation of percent HIV RNA^+^ cells and percent CD4^+^ T cells determined by flow cytometry. (G) Correlation of percent HIV RNA^+^ cells and the CD4/CD8 ratio. (H) Representative micrographs obtained by confocal microscopy of cells from an HIV-negative donor (left) and an untreated HIV-infected patient (right) after the RNA FISH-flow protocol. Staining of HIV RNA transcripts is red. All HIV RNA^+^ percentages were normalized to those of uninfected donors (obtained by background subtraction) for each independent experiment. White symbols correspond to values below the limit of detection. In panels D to G, values for untreated patients are represented by gray symbols and values for treated patients are represented by green symbols. *, *P* < 0.05; **, *P* < 0.01 (Mann-Whitney test). Spearman’s nonparametric correlation coefficients and associated *P* values are shown. Data for patients 1 to 6 and 25 to 40 are shown in panels B to F; data for patients 1 to 6, 28 to 33, and 36 to 39 are shown in panel G; and data for patient 26 are shown in panel H (Table S1).

We next determined the percentages of HIV RNA-expressing cells in unstimulated and unfractionated PBMC samples from patients with chronic untreated HIV infection and patients on ART (<20 copies/ml) compared to uninfected (HIV^−^) donors (patient characteristics are shown in Table S1). Representative flow cytometry strategy and raw data from different samples are shown in Fig. 2A and S3. When the percentage of CD4^+^ T cells expressing HIV RNA in 15 million PBMCs was analyzed, untreated HIV-infected patients showed statistically significantly larger proportions of cells transcribing HIV than did treated patients or healthy controls (median values of 0.0165 for untreated patients and 0.001 for treated patients) (Fig. 2B). Therefore, the frequency of cells transcribing HIV RNA from untreated patients was estimated at 165 per million CD4^+^ T cells, while in ART-treated individuals, we observed a frequency of 10 RNA-transcribing cells per million CD4^+^ T cells. These values are in agreement with previous reports (36). When we stratified the samples according to the patients’ plasma viral loads, we observed that more actively transcribing cells were consistently better detected in samples with high plasma viral loads. We were able to detect HIV transcripts in two out of six samples from treated aviremic HIV-infected individuals (Fig. 2C). For instance, the normalized values of HIV RNA-expressing cells showed a strong positive correlation with plasma viral loads (*r* = 0.823, *P* < 0.0001) (Fig. 2D) and negatively correlated with absolute CD4^+^ T cell counts (*r* = −0.723, *P* = 0.0001), percentages of CD4^+^ T cells in the original samples (*r* = −0.686, *P* = 0.0004), and CD4/CD8 ratios (*r* = −0.729, *P* = 0.002) (Fig. 2E to G). When we correlated the percentage of positive cells expressing HIV RNA with viral reservoirs markers, we observed a positive correlation with total HIV DNA (*r* = −0.672, *P* = 0.039) and intracellular levels of HIV RNA measured by conventional quantitative PCR (qPCR) (*r* = 0.721, *P* = 0.023) (Fig. S5A and B). The median frequency of HIV RNA-expressing cells obtained with the RNA flow/FISH assay was 80 events per million CD4^+^ T cells, compared to 12,804 molecules for total HIV DNA and 79,871 molecules for intracellular levels of HIV RNA per million CD4^+^ T cells measured by qPCR (Fig. S5C). In addition, when we performed the qVOA assay with samples from a separate cohort of aviremic individuals, we did not observe any correlation between the number of infectious units per million CD4^+^ T cells and the frequency of memory CD4^+^ T cells expressing HIV RNA obtained with the RNA flow/FISH assay (Fig. S5D). Overall, the RNA flow/FISH method measured approximately 3-log-fold fewer positive events than the conventional qPCR method. This difference is due to the fact that the two techniques measure different outcomes; the RNA flow/FISH method measures the percentage of HIV RNA-expressing cells, and detection of a positive event requires several RNA molecules per cell. On the contrary, the conventional qPCR method quantifies the number of RNA or DNA molecules in the whole population of CD4^+^ T cells containing both HIV-infected and uninfected cells. In this regard, confocal microscopy images of cells transcribing HIV showed numerous positive dots corresponding to HIV RNA molecules per individual cell (Fig. 2H). Additionally, in some primary PBMC samples from HIV-infected patients, we costained HIV RNA and the viral Gag p24 protein. Dual positive cell events were absent or very rarely detected in any population analyzed (data not shown). The assay of insufficient cell numbers might explain the failure to detect infected cells harboring both viral markers. Overall, this novel assay detects actively HIV RNA-transcribing cells in primary samples from infected individuals, and importantly, levels of cells expressing HIV RNA correlate very well with different markers of disease progression and intracellular levels of HIV RNA measured by conventional qPCR.

10.1128/mBio.00876-17.5 FIG S5  Correlations between the RNA FISH-flow technique and viral DNA and RNA quantification by conventional methods. (A) Spearman correlation between the percentage of HIV-expressing cells determined by the RNA FISH-flow assay and the total HIV DNA quantified by conventional qPCR. (B) Spearman correlation between the percentage of HIV-expressing cells determined by the RNA FISH-flow assay and intracellular HIV RNA quantification by qPCR. (C) Comparison of values obtained with the RNA FISH-flow assay, intracellular HIV RNA quantification by qPCR, and total HIV DNA quantification by qPCR. (D) Correlation between qVOA values and proportions of HIV RNA-expressing memory CD4^+^ T cells in samples from aviremic patients. Data for patients 3, 26, 27, 30, 32, 35, 36, and 38 to 40 are shown in panel A; those for patients 1, 3, 27, 30, 32, 35, 36, and 38 to 40 are shown in panel B; those for patients 1, 3, 26, 27, 30, 32, 35, 36, and 38 to 40 are shown in panel C; and those for patients 7, 10, 11, 16 to 20, and 24 are shown in panel D. Download FIG S5, EPS file, 1.3 MB. Copyright © 2017 Grau et al. 2017 Grau et al. This content is distributed under the terms of the Creative Commons Attribution 4.0 International license.

### Identification of CD4^+^ T cell subpopulations expressing HIV-1 RNA transcripts after *ex vivo* infection of unstimulated PBMCs.

**FIG 3:**
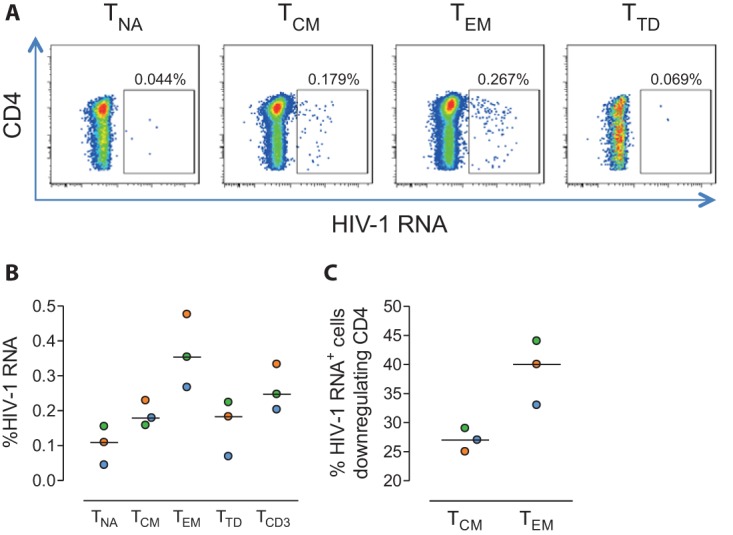
Detection of HIV RNA transcripts in different subsets of CD4^+^ T cells after *ex vivo* infection of PBMCs. Unfractionated and unstimulated PBMCs from healthy donors were infected *ex vivo* with exogenous HIV. Cells were subjected to the RNA FISH-flow protocol 5 days after infection. (A) Representative flow cytometry plots of HIV RNA-expressing cells in different subsets of CD4^+^ T cells (T_NA_, T_CM_, T_EM_, and T_TD_). (B) Quantification of HIV RNA^+^ cells in the different CD4^+^ T cell subsets of three different infected HIV-negative donors. (C) Percentages of HIV-expressing T_EM_ and T_CM_ cells that downregulate the CD4 receptor upon infection.

We then assessed the presence of HIV RNA transcripts in different CD4^+^ T cell subsets after *ex vivo* infection of unstimulated and unfractionated PBMCs from healthy donors. Cells were first infected with an exogenous virus and then stained with antibodies specific for the markers CD3, CD4, CD45RO, and CCR7 to identify different subpopulations of CD4^+^ T cells. The percentages of HIV RNA-positive cells within the naive (T_NA_), central memory (T_CM_), effector memory (T_EM_), and terminally differentiated (T_TD_) CD4^+^ T cell subsets of a representative *ex vivo* infection experiment are shown in Fig. 3A. The expression of HIV RNA transcripts was observed mainly in T memory cells (~0.2% of the total cell population) (Fig. 3A). Specifically, the majority of cells supporting HIV transcription had a T_EM_ CD4^+^ T cell phenotype and represented >50% of all infected cells (Fig. 3B). The percentage of T_EM_ cells expressing HIV RNA was higher than in the whole T cell (T_CD3_) population (median proportion of positive cells of 0.35 for T_EM_ cells and 0.24 for CD3^+^ T cells) (Fig. 3B), indicating an enrichment of the subpopulation that supports most active transcription of HIV (1.38-fold enrichment in T_EM_ cells compared to T_CD3_ cells). Moreover, infected cell subpopulations only partially downregulated the CD4 cell receptor upon infection, with the T_EM_ subset being the subpopulation sustaining greater downregulation of the CD4 marker (~40% of all T_EM_-infected cells) (Fig. 3C).

### Phenotypic characterization of CD4^+^ T cells expressing HIV-1 RNA transcripts in primary samples from HIV-infected patients.

**FIG 4:**
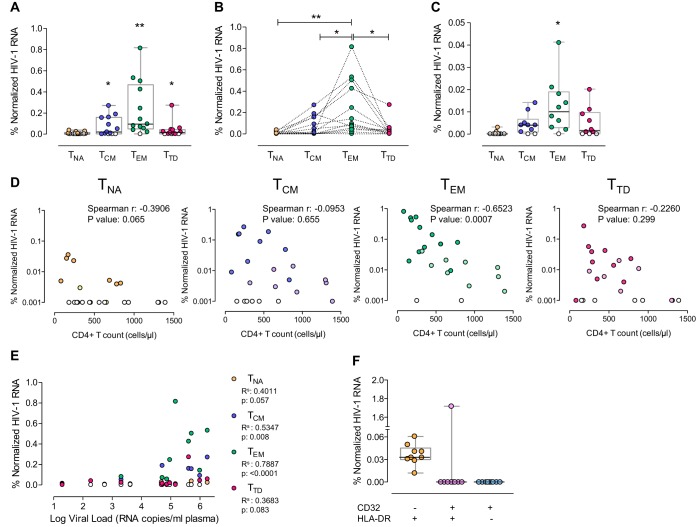
Identification of CD4^+^ T cell subsets supporting HIV expression in primary samples from HIV-infected patients. Fifteen million unfractionated and unstimulated PBMCs from HIV-infected patients were subjected to the RNA FISH-flow protocol for viral transcript detection in the different CD4^+^ T cell subsets (T_NA_, T_CM_, T_EM_, and T_TD_). (A) Frequencies of HIV RNA-expressing cells in different CD4^+^ T cell subpopulations of untreated patients (*n* = 13). The frequency of viral transcription in each subpopulation was compared with that of the HIV-negative control by using the Mann-Whitney test. (B) Paired comparison of HIV RNA^+^ cells in CD4^+^ T cell subsets of untreated patients (*n* = 13). The statistical values shown were obtained with the Wilcoxon signed-rank test with corrected *P* values for multiple comparisons. (C) Frequency of HIV RNA-expressing cells in CD4^+^ T cell subsets of treated patients with undetectable plasma viral loads (*n* = 10). (D) Spearman correlation of HIV RNA frequency in the different CD4^+^ T cell subpopulations and the absolute CD4^+^ T cell counts (*n* = 23). (E) Spearman correlation between plasma viral loads and the percentages of CD4^+^ T cells expressing HIV RNA in different subsets (*n* = 23). For all correlations, white symbols represent values below the limit of detection, symbols representing values for untreated patients are in dark colors, and symbols representing values for treated patients are in pale colors. (F) Frequencies of HIV RNA-expressing cells in CD4^+^ T cells expressing CD32 and HLA-DR. All normalized values were obtained after subtracting the corresponding background signal observed in the HIV-negative control. *, *P* < 0.05; **, *P* < 0.01. Data for patients 28 to 40 (Table S1) are shown in panels A and B; those for patients 1 to 6, 10, and 13 to 15 are shown in panel C; those for patients 1 to 6, 10, 13 to 15, and 28 to 40 are shown in panels D and E; and those for patients 7, 10, 11, 16 to 20, and 24 are shown in panel F.

After the successful identification of the CD4^+^ T cell subpopulations transcribing HIV in *ex vivo*-infected samples, we sought to phenotypically characterize the subsets of CD4^+^ T cells that support HIV transcription *in vivo*. Fifteen million unfractionated and unstimulated PBMCs from untreated HIV-infected patients were subjected to the RNA FISH-flow assay to determine the frequency of HIV RNA-expressing cells. A representative gating strategy used to identify T cell subsets in an HIV-infected patient is shown in Fig. S6A. We observed greater proportions of HIV-transcribing cells in all of the subsets assayed than in uninfected controls, except naive T cells (median values: T_NA_, 0; T_CM_, 0.02; T_EM_, 0.095; T_TD_, 0.014) (Fig. 4A). Consistent with the *ex vivo* infection experiments, the T_EM_ subset was dominant among the HIV RNA-expressing cells and the proportion of cells expressing HIV was greater in T_EM_ cells than in all other CD4^+^ T cell subsets (fold increases in T_EM_ versus other subsets: 124.7 for T_NA_, 24.8 for T_CM_, 108.44 for T_TD_) (Fig. 4B). In addition, HIV RNA-expressing cells were detected significantly more often within the T_EM_ subset in samples from aviremic treated HIV-infected patients than in uninfected control samples (Fig. 4C). Moreover, the percentage of T_EM_ cells expressing viral transcripts was also higher than that of the other assayed CD4^+^ T cell subsets. We next assessed the correlation of HIV RNA-expressing cells in the different T cell subsets by using plasma viral loads and CD4^+^ T cell counts as progression markers. There was a positive correlation between the plasma viral loads and the percentages of T_CM_ and T_EM_ HIV RNA-expressing cells, although a more robust correlation was observed for T_EM_ cells (*r* = 0.7887, *P* < 0.0001) (Fig. 4E). In contrast, we observed a strong inverse correlation between HIV-expressing T_EM_ cell and CD4^+^ T cell counts (*r* = −0.652, *P* = 0.0007) (Fig. 4D), similar to what was observed in the entire CD4^+^ T cell compartment (Fig. 2D). Additionally, after the identification of CD32 as a marker of latently infected cells (11), we wanted to investigate if cells expressing HIV in samples from aviremic patients were also expressing the CD32 marker. We observed that cells expressing the CD32 receptor represented only a small proportion of the total CD4^+^ T cells (median, 0.063; interquartile range, 0.060 to 0.104). Only one of the patients analyzed contained HIV RNA-expressing cells in this population of cells (Fig. 4F). Taken together, these results indicate that we successfully detected different subpopulations of cells expressing HIV RNA with the flow RNA FISH protocol. Moreover, we observed that the effector memory T cell subset was largely responsible for the production of HIV RNA in CD4^+^ T cells in both treated and untreated HIV-infected patients.

10.1128/mBio.00876-17.6 FIG S6  Representative example of the flow cytometry gating strategy used for the identification of CD4^+^ T cell subsets in patient samples. (A) Subpopulations are defined as T_NA_ (CD3^+^ CD4^+^ CD45RO^−^ CCR7^+^) T_CM_ (CD3^+^ CD4^+^ CD45RO^+^ CCR7^+^), T_EM_ (CD3^+^ CD4^+^ CD45RO^+^ CCR7^−^), and T_TD_ (CD3^+^ CD4^+^ CD45RO^−^ CCR7^−^). (B) Gating strategy used for the identification of HIV-expressing cells in different CD4^+^ T cell populations based on the expression of CD32 and HLA-DR. Download FIG S6, EPS file, 4.1 MB. Copyright © 2017 Grau et al. 2017 Grau et al. This content is distributed under the terms of the Creative Commons Attribution 4.0 International license.

### Detection of viral reactivation in primary CD4^+^ T cells from ART-treated HIV-infected patients.

**FIG 5:**
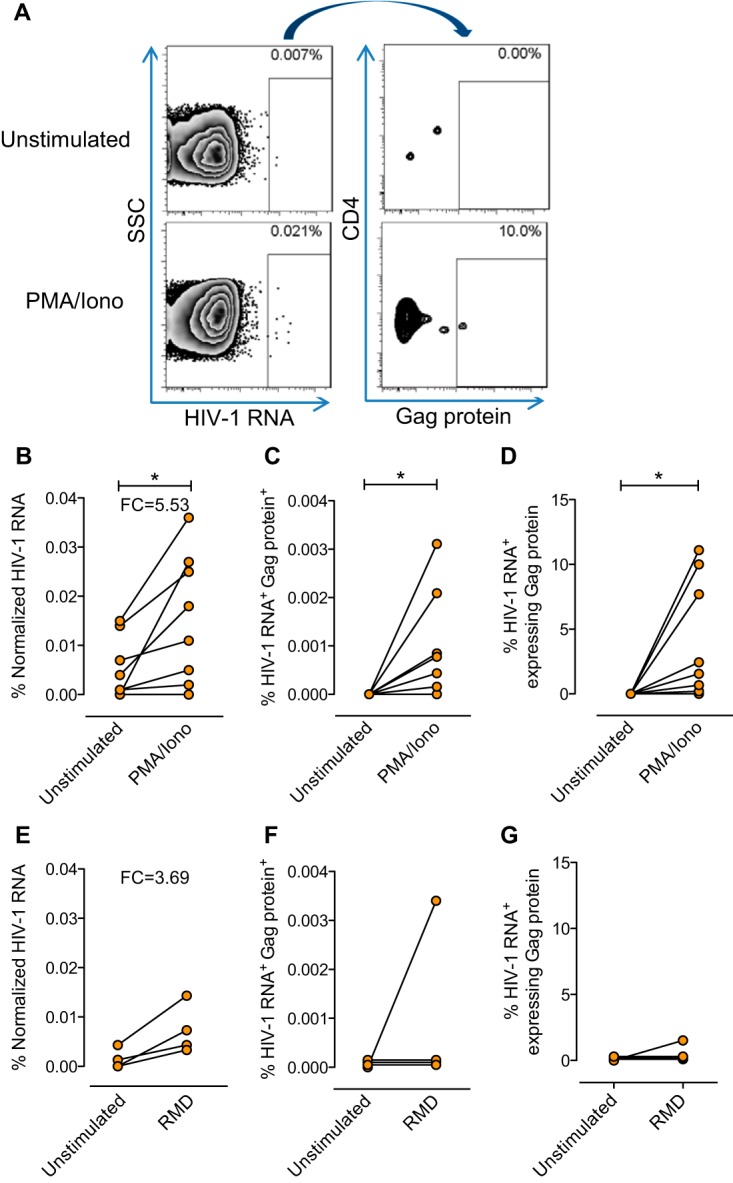
Detection of HIV-1 RNA and Gag p24 protein in primary CD4^+^ T cells from HIV-infected patients after *ex vivo* reactivation. CD4^+^ T cells from treated HIV-infected patients were isolated with magnetic beads. Expression of HIV RNA and the viral Gag p24 protein was detected by the RNA FISH-flow technique after 13 h of reactivation with PMA-Iono or 24 h of reactivation with romidepsin. (A) Representative flow cytometry plots of cells from a treated patient before and after viral reactivation with PMA-Iono. Live cells were identified, and single HIV RNA-expressing cells gated against side scatter (SSC) are shown on the left. Percentages of HIV RNA^+^ cells that were Gag p24^+^ are shown on the right. (B, C) Comparisons of HIV-1 RNA^+^ single cells after viral reactivation with PMA-Iono (B) and HIV-1 RNA^+^ Gag^+^ doubly positive cell frequencies before and after viral reactivation (PMA-Iono) (C). (D) Frequencies of HIV-1 RNA^+^ single cells expressing Gag^+^ before and after stimulation with PMA-Iono. (E) Comparison of HIV-1 RNA^+^ single cells after viral activation with romidepsin. (F, G) Comparisons of HIV-1 RNA^+^ single cells after viral reactivation with romidepsin (F) and doubly positive cells HIV-1 RNA^+^ Gag^+^ frequencies before and after viral reactivation (romidepsin) (G). Cells from nine patients were treated with PMA-Iono (patients 5 and 7 to 13 are shown in panels B to D), and cells from four patients were treated with romidepsin (RMD) (patients 7 and 21 to 23 are shown in panels E to G). *, *P* < 0.05 (paired nonparametric *t* test). FC, fold change.

Finally, we assessed the ability of this assay to detect HIV RNA transcripts and the Gag p24 viral protein from previously isolated CD4^+^ T cells after *ex vivo* viral reactivation. We evaluated primary samples from treated aviremic infected patients and assessed the capacity of a strong non-HIV-specific activating stimulus (phorbol 12-myristate 13-acetate [PMA]–ionomycin [Iono]) and romidepsin, a histone deacetylase inhibitor, to induce the transcription and translation of HIV. A representative flow cytometry plot is shown is Fig. 5A. Overall, we observed an increase in the percentage of cells expressing HIV RNA upon cell activation with PMA-Iono compared with the corresponding control (5.53-fold change), and upon cell activation with romidepsin, four out of four patients showed increasing proportions of HIV RNA-expressing cells (3.69-fold change) (Fig. 5B and E). The median numbers of RNA-positive cells per million CD4^+^ T cells before and after viral reactivation were 20 and 145 for PMA-Iono and 6 and 58 for romidepsin, respectively. Moreover, the expression of cells doubly positive for HIV RNA and Gag p24 rose significantly when cells were stimulated with PMA-Iono, with an estimated median of six cells with inducible HIV per million CD4^+^ T cells (Fig. 5C). For romidepsin, we observed positive p24 events in only one patient (Fig. 5F). Of note, the range of dually positive cells was highly dependent on the patient. Also, when we analyzed the percentage of cells transcribing HIV RNA that also translated Gag p24 after stimulation with PMA-Iono, we observed a significant increase in the numbers of HIV RNA^+^ cells concomitantly expressing HIV proteins. However, the degree of expression was also highly variable between different patients and was observed only for PMA-Iono (Fig. 5D). For romidepsin, however, this increase was very modest and was observed in only one patient (Fig. 5G). Thus, this assay could be used to evaluate the effectiveness of different LRAs in reversing the latent state of the HIV reservoir in different cell populations.

## DISCUSSION

Identification of the HIV-1 cell reservoirs that exist in HIV-infected patients will significantly advance the design of new targeted therapies aimed at curing HIV. In this regard, the identification and characterization of active viral reservoirs, defined as infected cells with transcriptionally active HIV, have been limited by an inability to isolate the required amount of cells for accurate quantification of viral transcription. In this investigation, we used a new RNA FISH-flow assay for the identification of HIV-infected cells that express viral transcripts both after *ex vivo* infection and in primary samples from HIV-infected patients. In addition, we used this novel method to assess viral reactivation in primary CD4^+^ T cells from HIV-infected patients after *ex vivo* exposure to an activating stimulus.

Using this novel assay that measures transcriptionally competent HIV-infected cells, we found that after *ex vivo* infection of unstimulated PBMCs, HIV transcription was significantly detected in T cells and production of viral proteins correlated well with the downregulation of the CD4 cell receptor. This finding is in agreement with previously published data showing that different viral proteins are responsible for the downregulation of CD4 on the surface of the infected cell (38, 39) and higher levels of HIV RNA expression are required for productive infection (40, 41). Importantly, we also observed that non-T cells expressed HIV transcripts and viral proteins and most of these non-T cells expressed the CD4 receptor. In this investigation, we have not fully identified these non-T-cell populations harboring both HIV RNA and the Gag p24 protein, although viral capture by dendritic cells and viral replication in monocytes would be a possible explanation for the present results (42, 43). More precise phenotypic studies are needed to fully identify the non-T-cell populations supporting HIV transcription and translation after *ex vivo* infection.

Using the RNA flow/FISH protocol, we found that the majority of HIV RNA-positive cells in primary samples from infected patients were detected in CD4^+^ T cells and we were not able to observe significant detection of HIV transcripts in the CD4^−^ T cell population. This result is consistent with previous reports where CD4^+^ T cells were enriched in unspliced viral transcripts compared to T cells that downregulated the CD4 receptor (44). Moreover, in treated patients, HIV RNA in blood and tissue was highly concentrated in the CD4^+^ population compared to non-CD4^+^ T cells (45). In addition, T cells that downregulate the CD4 receptor support the greatest production of viral particles; thus, higher rates of cell death in this infected population during the experimental protocol might also account for our results (46). In agreement with the lack of significant detection of HIV RNA in CD4^−^ T cells, we did not detect cells expressing both viral RNA and the viral Gag p24 protein in samples from HIV-infected patients without previous cell stimulation. In addition to cell death, the assay of insufficient cell numbers may explain the lack of detection of dually stained cell populations.

Frequencies of CD4^+^ T cells expressing HIV transcripts in samples from HIV-infected patients measured by this novel method had a median of 10 positive cells per million CD4^+^ T cells in aviremic patients and 165 positive cells per million CD4^+^ T cells in viremic untreated individuals. These data fully agree with previous studies (29, 36). Importantly, these frequencies correlated very well with different markers of disease progression such as plasma viral loads, CD4^+^ T cell counts, and CD4/CD8 ratios. Our data agree with previously published data showing that intracellular HIV RNA quantification strongly correlated with disease progression and inversely correlated with CD4^+^ T cell counts in untreated viremic patients (30–33). Previous reports have shown that during the chronic phase of infection, intracellular levels of HIV RNA range between 3- and 4-log magnitudes of molecules per million unfractionated PBMCs (31); however, after ART initiation, the levels of cell-associated HIV RNA only decline about 1 to 2 logs (47, 48) and positively correlate with the total viral reservoir measured by total HIV DNA quantification (49). When we performed a direct comparison of the quantitative values obtained by conventional methods of intracellular viral nucleic acids and the percentage of positive cells measured by the RNA FISH-flow technique, we observed a direct significant correlation between the two methods; however, our novel method quantified 3-log-fold lower values than the RNA molecule levels in CD4^+^ T cells quantified by qPCR and 2-log-fold lower values than the number of DNA molecules per million CD4^+^ T cells measured by conventional methods. The impossibility of measuring the exact numbers of HIV RNA and DNA molecules in a single cell by qPCR makes this comparison biased, since detection of positive events by the RNA FISH-flow technique most likely requires the presence of several HIV RNA molecules, as we have observed in our micrographs. In this regard, previous reports showed that productively HIV-infected cells contained ~300 HIV RNA copies per cell (35, 50, 51). Moreover, the lower sensitivity of the RNA FISH-flow method than the PCR-based method might also explain part of these differences. When we compared the proportions of memory CD4^+^ T cells expressing HIV RNA calculated by the RNA FISH-flow assay and the qVOA, we did not observe any correlation. The proportion of viral RNA-expressing cells not able to produce fully replicative virions might be responsible for this result. In the same line, intracellular HIV RNA quantification by qPCR has correlated poorly with qVOA results in previous studies (17, 18).

In treated aviremic patients, the identification of the cellular sources of HIV transcripts is of particular importance since this information will directly identify the specific cell subpopulations supporting HIV persistence. HIV transcripts can be found in latently infected cells that do not produce viral particles, in productive infection of new cells, or in cells reactivated from latency with productive production of viral particles (26). To date, the precise composition of the cellular subpopulations that support this active viral reservoir in HIV-infected patients is largely unknown. We identified the effector memory CD4^+^ T cell subpopulation as the main subset of cells expressing HIV during both untreated and treated infections. In this regard, the detection of infected cells in the fraction of PBMCs could be an intrinsic limitation of our study, since central memory T cells recirculate constitutively between secondary lymphoid organs and blood and effector memory T cells circulate through blood and home to inflamed tissue (52). Thus, not all of the cell subsets analyzed in this study are continuously present in peripheral blood. Of note, identification of effector memory CD4^+^ T cells was carried out by using the phenotypic markers CD45RO and CCR7, and therefore, this population can also contain cells with a transitional memory phenotype (53).

In agreement with our data, previous studies identified effector memory cells as the main subset supporting HIV transcription in both peripheral blood and ileum and rectum tissue samples from ART-treated patients (45). On the basis of HIV DNA quantification, T_EM_ cells were also identified as one of the main subsets harboring total and integrated HIV DNA (22, 23, 25). Follicular helper T cells have been recently identified as one of the main cell subsets that support the replication, transcription, and production of HIV-1 (24). Although we did not perform a detailed investigation of that specific cell subpopulation in this study, we present our method as a promising tool for the identification of transcriptionally active HIV in cell subpopulations, including follicular helper T cells, from different tissue samples.

A new biomarker of latently infected cells has recently been described. Descours et al. reported that the immunoglobulin G Fc fragment CD32a was exclusively induced in quiescent infected cells. Productive infection of stimulated CD4^+^ T cells was not associated with significant CD32a expression, compared to that in resting CD4^+^ T cells (11). Thus, we also explored the expression of CD32 in cells transcribing HIV *in vitro* and *in vivo* by our newly described method. We found that after *ex vivo* infection of unstimulated PBMCs, infected cells concomitantly upregulated the expression of CD32 and the activation marker HLA-DR; productively infected cells expressing HIV RNA and Gag protein upregulated both markers to a greater degree (2-fold increase) than cells transcribing only HIV RNA (1.5-fold increase). Moreover, the proportion of cells expressing CD32 also increased. On the basis of these results, we concluded that productive HIV infection upregulates the expression of CD32. Levels of CD32 were very modest compared to those of non-T cells. Of note, with our system, we cannot compare these levels to those expressed in latently infected cells. The discrepancy between the study of Descours et al. and our results may be due to the fact that Descours et al. stimulated their PBMCs before productive infection, while we performed infections without any previous cell stimulation. In addition, Descours et al. focused their analysis on the HLA-DR^−^ cell population. Further studies are certain to fully elucidate the role of CD32 in HIV infection and latency.

HIV RNA transcription is a useful tool with which to assess the effect of LRAs in new therapeutic approximations aimed at curing HIV. LRAs are being used in clinical trials to reactivate HIV from its dormant state. However, clinical studies have shown only a modest reduction of the latent HIV reservoir after LRA administration (15, 16, 54). As LRAs may not work equally in all infected cell subpopulations, new methods to assess the effectiveness of LRAs *in vitro* are highly desirable. Previous reports have shown the ability of the RNA FISH-flow assay to detect viral reactivation in primary samples and cell lines (55, 56). Here, we used our method to detect viral reactivation in primary infected CD4^+^ T cells after the *ex vivo* administration of an activating stimulus. In samples from treated HIV-infected patients, romidepsin significantly increased the percentage of cells expressing HIV RNA; however, we were not able to detect a significant production of viral proteins. This result is consistent with previously reported studies where romidepsin alone failed to induce robust production of viral particles (57, 58). However, upon cell stimulation with PMA-Iono, significant percentages of cells expressing HIV and viral proteins were successfully detected. HIV RNA expression increased very modestly upon viral reactivation. The fact that we focused our analysis on cells with high expression of HIV RNA to avoid an increased background might account for this result. Importantly, and in agreement with our data, previous reports have shown that the number of unspliced HIV RNA molecules per cell during different stages of viral production varies only slightly; transcription of unspliced HIV RNA per cell was unaffected by treatment status and ongoing viral replication (35, 36). Using this method, we estimated that the median size of the inducible reservoir, defined as cells able to express viral RNA and the Gag p24 protein upon cell activation, was six per million CD4^+^ T cells. This number is higher than previously reported frequencies of latently infected cells measured by the gold standard qVOA assay (12, 13). However, we are not able to exclusively detect the fraction of cells with replication-competent virus. Our estimated size of the inducible reservoir is, however, smaller than that predicted by the *tat*/*rev*-induced limiting-dilution assay, which accounts for the fraction of cells with transcriptionally active HIV-1 (29). How our novel method correlates with previously reported assays designed to measure the size of the latent reservoir deserves a more detailed investigation; in-depth comparison with the same samples will be indispensable to fully estimate the ability of the RNA flow/FISH assay to accurately measure the size of the HIV-1 reservoir.

An assay that detects translation-competent HIV-infected cells on the basis of the same technology has been recently reported (56). Baxter et al. simultaneously detected both HIV RNA and the viral Gag p24 protein in primary HIV-infected samples. However, a very high background signal level in the HIV RNA channel precluded the use of the assay to identify the fraction of transcriptionally competent HIV-infected cells. In our study, however, the use of a high-sensitivity set of 50 probes that recognize the Gag-Pol sequence of the HXB2 consensus genome with a minimized background signal and subtraction of the nonspecific background from the quantification of HIV RNA-expressing cells allowed us, for the first time, to detect significant differences in the levels of HIV RNA-expressing cells in different cohorts of HIV-infected patients.

Overall, in this study we have found that effector memory CD4^+^ T cells are an important niche for HIV transcription *in vivo*. The identification of transcriptionally active HIV-infected cells will provide important information on the specific cellular and anatomical sources of residual viremia, the contribution of HIV to viral pathogenesis, and the specific cellular reservoirs that persist in ART-treated individuals. In addition, this novel method detects viral reactivation in primary CD4^+^ T samples, providing a useful tool with which to evaluate the effectiveness of different LRAs in different cell subpopulations.

## MATERIALS AND METHODS

### Ethics statement.

PBMCs from HIV-1-infected patients were obtained from the HIV unit of the Hospital Universitari Vall d’Hebron in Barcelona, Spain. Written informed consent was provided by all of the patients who participated in this study, and the protocols used were approved by the Comité d’Ètica d’Investigació Clínica (Institutional Review Board numbers 39-2016 and 196-2015) of the Hospital Universitari Vall d’Hebron, Barcelona, Spain. Samples were obtained only from adults, who all provided written informed consent, and the samples were prospectively collected and cryopreserved in the Biobanc (register number C.0003590). All samples received were totally anonymous and untraceable. The J-Lat cell line and the T-lymphoblastoid MOLT-4 CCR5+ cell line were obtained from the NIH AIDS Reagent Program.

### Study samples.

Samples from HIV-1-infected patients with CD4^+^ T cell counts of >100/mm^3^ in the HIV unit of the Hospital Universitari Vall d’Hebron in Barcelona, Spain, were included in this study. Information on plasma viral loads, CD4^+^ T cell counts, and time on ART for treated patients is summarized in Table S1.

### Cells and virus.

PBMCs were obtained from HIV-1-infected patients by Ficoll-Paque density gradient centrifugation and cryopreserved in liquid nitrogen. PBMCs from healthy donors were obtained anonymously from the BST (Banc de Sang I Teixits, Barcelona, Spain) and isolated as described above.

The human latently infected cell line J-Lat (clone 9.2) was obtained through the NIH AIDS Reagent Program from Eric Verdin (59); grown in RPMI 1640 medium supplemented with 10% fetal bovine serum (FBS; Gibco, Life Technologies, Inc.), 100 U/ml penicillin, and 100 µg/ml streptomycin (Life Technologies, Inc.); and maintained at 37°C in a 5% CO_2_ incubator. The T-lymphoblastoid MOLT-4 CCR5+ cell line (obtained through the NIH AIDS Reagent Program from Masanori Baba, Hiroshi Miyake, and Yuji Iizawa) (60) was cultured in R10 (RPMI medium with 10% FBS) containing 1 mg/ml G-418.

The plasmid encoding HIV-1 strain NL4.3 (pNL4.3) was obtained through the NIH AIDS Reagent Program from Malcom Martin. Viral stocks were generated by transfection of 293T cells with pNL4.3, and the resulting viral particles were titrated in TZMbl cells by enzyme luminescence assay (britelite plus kit; PerkinElmer) as described previously (61).

### *Ex vivo* infection of unstimulated PBMCs.

PBMCs from healthy donors were quickly thawed and incubated overnight in R10 with 100 µg/ml streptomycin, 100 U/ml penicillin, and 40 U/ml interleukin-2 (IL-2). On the next day, PBMCs were infected (350,000 50% tissue culture infective doses/million cells) with the NL4.3 viral strain for 4 h at 37°C and 5% CO_2_. Following the initial infection, the cells were thoroughly washed and cultured in six-well plates in R10 plus 100 U/ml IL-2 for an additional 5 days to expand the viral infection. The cells were then subjected to the RNA FISH-flow protocol as described below.

### Detection of single cells expressing HIV-1 RNA transcripts by the RNA FISH-flow assay.

The RNA FISH-flow assay for detection of HIV transcripts was performed in accordance with the manufacturer’s instructions (Human PrimeFlow RNA Assay; EBioscience), with some modifications. Briefly, 15 million PBMCs were stained with antibodies to cell surface markers (20 min at room temperature [RT]; CCR7, 30 min at 37°C) and violet viability dye (20 min at RT). Cells were then fixed, permeabilized, and intracellularly stained for detection of the viral p24 protein when required (60 min at 4°C). After an additional fixation step, cells were ready for 3 h of hybridization at 40 ± 1°C with a high-sensitivity target-specific set of 50 probes spanning the whole Gag-Pol HIV mRNA sequence (bases 1165 to 4402 of the HXB2 consensus genome). The cells were then subjected to different amplification steps (sequential 2-h incubations at 40°C with the preamplification and amplification mixtures). Finally, multiple label probes were hybridized with the specific amplifiers (1 h at 40°C). Negative controls were included in all experiments with cells from non-HIV-infected donors. The normalized percentage of HIV RNA expression was calculated for each subpopulation by subtracting the mean value obtained from the negative control from the signal obtained with the real sample. All samples were run on an LSR Fortessa four-laser flow cytometer (Becton, Dickinson).

### Antibody panel.

To identify the different CD4^+^ T cell subpopulations expressing HIV RNA, PBMCs were stained for cell surface markers with CD4 (AF700; BD), CD3 (phycoerythrin [PE]-Cy7; BD), CCR7 (PE; BD), and CD45RO (BV605; BioLegend) antibodies. The CD4^+^ T cell subset phenotypes were identified as follows: T_NA_, CD3^+^ CD4^+^ CCR7^+^ CD45RO^−^; T_CM_, CD3^+^ CD4^+^ CCR7^+^ CD45RO^+^; T_EM_, CD3^+^ CD4^+^ CCR7^−^ CD45RO^+^; T_TD_, CD3^+^ CD4^+^ CCR7^−^ CD45RO^−^. For detection of CD32 after *ex vivo* infection, we used CD4 (AF700; BD), CD3 (PE-Cy5; BD), p24 (PE; Beckman Coulter, Inc.), CD32 (PE-Cy7; BioLegend), and HLA-DR (fluorescein isothiocyanate; BioLegend) antibodies. For detection of CD32 in samples from HIV-infected patients, we used CD4 (AF700; BD), CD3 (PE-Cy5; BioLegend), CD45RO (BV605; BioLegend), CD32 (PE-Cy7; BioLegend), and HLA-DR (PE; BioLegend) antibodies. The expression of HIV RNA transcripts was analyzed with target-specific AF647-labeled probes, and expression of the viral Gag p24 protein was detected with a PE-conjugated anti-p24 antibody (clone KC57 RD1; Beckman Coulter, Inc.). Cell viability was determined with a violet viability dye for flow cytometry (LIVE/DEAD Fixable Violet Dead Cell Stain kit; Invitrogen).

### Linearity of the RNA FISH-flow assay.

To test the linearity of the assay, latently infected Jurkat (J-Lat clone 9.2) cells were spiked with the T-lymphoblastoid MOLT-4 CCR5+ cell line at four different ratios (1/3 serial dilutions). Samples were then subjected to the RNA FISH-flow assay. The predictive curve was determined by the basal expression of green fluorescent protein within the J-Lat cells and the subsequent theoretical values of the serial dilutions. The infection rate (experimental curve of percent HIV RNA^+^ cells) was calculated by using the values obtained with the RNA FISH-flow assay. Linear regression was computed to determine the linearity of the relationship between the predicted and experimental values of the assay. The linearity of the assay was also measured after expanding the infection of primary CD4^+^ T cells from HIV-infected patients. We used the same protocol described for the qVOA assay, and the positive wells were mixed up and diluted into uninfected cells at six different ratios. The predictive curve was determined by the basal expression of p24 and the subsequent theoretical values of the serial dilutions.

### *Ex vivo* viral reactivation of primary CD4^+^ T cells.

Primary CD4^+^ T lymphocytes from PBMCs of HIV-infected patients were purified by negative selection in accordance with the instructions provided by the manufacturer (MagniSort Human CD4^+^ T Cell Enrichment; Affymetrix). Isolated CD4^+^ T cells (purity routinely >95%) were cultured in complete RPMI medium supplemented with 10% FBS, 100 µg/ml streptomycin, and 100 U/ml penicillin alone or stimulated with PMA (50 ng/ml; Abcam, Inc.) and Iono (0.5 µg/ml, Abcam, Inc.) for 13 h in the presence of raltegravir (1 µM) and nevirapine (100 nM) or with romidepsin (40 nM) for 24 h. Cells were then extensively washed and subjected to the RNA FISH-flow assay for detection of viral RNA and the viral Gag p24 protein. A minimum of 2 million CD4^+^ T cells were used per condition.

### Confocal microscopy of cells following the RNA FISH-flow assay.

Following the RNA FISH-flow assay protocol, samples were fixed for 30 min at 4°C with 4% PFA, washed with phosphate-buffered saline, and smeared onto a microscope slide. Preparations were finally mounted with Fluoromount G (EBioscience). Preparations were imaged with an Olympus FV1000 Spectral Deconvolution Confocal Microscope. ImageJ software was used for all image compositions.

### HIV-1 RNA *in situ* hybridization in lymph nodes.

To evaluate the specificity of the target-specific high-sensitivity probes that detect HIV transcripts in primary samples from an HIV-infected patient (probes for bases 1165 to 4402 of the HXB2 consensus genome), we used the ultrasensitive ViewRNA ISH Tissue 2-Plex Assay kit (eBioscience), which detects RNA molecules in tissue samples. A lymph node sample from an acutely HIV-infected patient (patient 41, Table S1) was fixed and embedded in paraffin. Sections (6 µm each) were mounted on Superfrost Plus microscope slides (Fisher Scientific). Before the assay was performed, samples were heated for 1 h at 60°C, dewaxed in xylene, and then placed in 100% ethanol before being air dried. The ViewRNA protocol was performed in accordance with the manufacturer’s instructions. Briefly, heat-induced epitope retrieval was performed by boiling sections in pretreatment solution for 10 min, and additional protease digestion was performed at 40°C for 20 min. Hybridization was carried out by slide incubation with target probes for 2 h at 40°C. After that, samples were stored overnight in storage buffer. On the next day, signal amplification was achieved by sequential slide incubation with PreAmplifier Mix and Amplifier Mix for 25 and 15 min at 40°C, respectively. Samples were then incubated with the appropriated label probe and substrate. Finally, slides were counterstained with 4',6-diamidino-2-phenylindole (DAPI), air dried, and mounted with Fluoromount G. Samples were imaged on an Olympus FV1000 Spectral Deconvolution Confocal Microscope with a 10× phase objective in sequential mode to separately capture the fluorescence from the different fluorochromes at an image resolution of 800 by 800 pixels.

### HIV DNA and intracellular HIV RNA quantification by qPCR.

CD4^+^ T cells were isolated by negative selection as mentioned above. A total of 1.5 million cells were subjected to RNA extraction by the total RNA extraction protocol (mirVana; Ambion). RNA was reverse transcribed with SuperScriptIII (Invitrogen) in accordance with the instructions provided by the manufacturer, and cDNA was quantified by qPCR with primers and probes specific for the HIV long terminal repeat-gag region. Copies of HIV RNA were quantified with an HIV RNA standard, and values were normalized to micrograms of RNA of the original sample. For HIV DNA quantification, 1 million CD4^+^ T cells were immediately lysed with a proteinase K-containing lysis buffer. Cell lysates were subjected to total HIV DNA quantification as previously described (18). The gene for CCR5 was used for cell input normalization.

### qVOAs.

qVOAs were performed as previously described, with some modifications (18). Briefly, CD4^+^ T cells from HIV-1-infected patients were isolated by negative selection (Affymetrix) and cultured at 50,000/well in R10 supplemented with 10% FBS, 100 U/ml penicillin, and 100 µg/ml streptomycin. Subsequently, cells were stimulated with phytohemagglutinin (PHA; 2 µg/ml), recombinant IL-2 (100 U/ml), and irradiated allogeneic PBMCs (50 Gy in a Cs source irradiator for 20 min) obtained from HIV-negative healthy donors (100,000/well) and cultured in an incubator at 37°C and 5% CO_2_. After 48 h, the PHA was completely washed away and MOLT-4 CCR5+ cells were added at 10,000/well on day 2 of culture and again on day 9. The cultures were subjected to the removal of 100 µl of just medium on day 6 and 100 µl of the cell suspension on day 9 and replenished with fresh complete medium containing recombinant human IL-2 (100 U/ml). After 12 days, cell supernatant was collected from each well and the number of wells containing infectious HIV-1 was assessed by incubation of the supernatant with TZM-bl cells. Luciferase activity was quantified on day 14 by luminescence assay in accordance with the manufacturer’s instructions (britelite plus kit; PerkinElmer) and is directly proportional to the number of infectious virus particles present in the initial inoculum. Latently HIV-1-infected ACH-2 cells were run as positive controls. Estimated frequencies of cells with replication-competent HIV-1 were calculated by limiting-dilution analysis as described in reference 62.

### Statistical analysis.

Statistical analyses were performed with the Prism software, version 5.0 (GraphPad). Comparisons of the frequencies of HIV RNA-expressing cells in infected patients and healthy donors were performed with the nonparametric Mann-Whitney test. For correlations, Spearman’s correlation coefficient was calculated. To test the linearity of the assay, a linear regression was performed. A paired *t* test (Wilcoxon signed-rank test) was used to compare HIV RNA expression levels in CD4^+^ T cell subsets with corrected *P* values for multiple comparisons. A *P* value of <0.05 was considered significant.
