# Supplementary figures and images for: A Novel Single-Cell FISH-Flow Assay Identifies Effector Memory CD4+ T cells as a Major Niche for HIV-1 Transcription in HIV-Infected Patients
Source: mBio. 2017 Jul 11;8(4):e00876-17. doi: 10.1128/mBio.00876-17 (PMC5513707; doi:10.1128/mBio.00876-17)

Fig S1

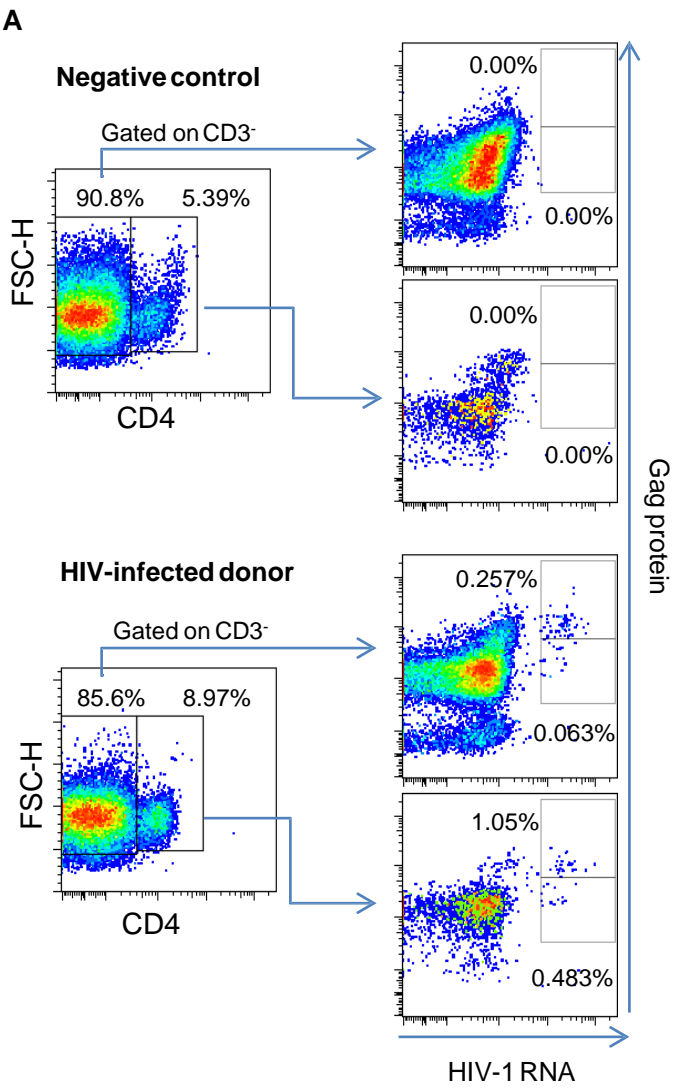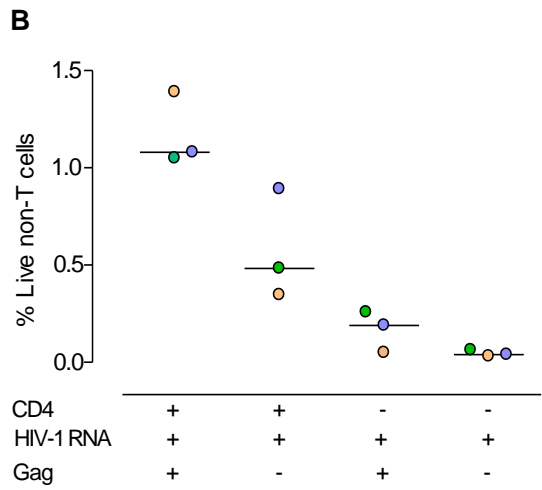

Supplement: FIG S1 [file mbo003173382sf1.pdf]
