# Supplementary material for: A Novel Single-Cell FISH-Flow Assay Identifies Effector Memory CD4+ T cells as a Major Niche for HIV-1 Transcription in HIV-Infected Patients
Source: mBio. 2017 Jul 11;8(4):e00876-17. doi: 10.1128/mBio.00876-17 (PMC5513707; doi:10.1128/mBio.00876-17)
Supplement: TABLE S1 [file mbo003173382st1.docx]

**Table S1.** Clinical data of patients included in the study.

| Patient ID | Time since HIV diagnosis (months) | CD4 Cell Count (cells/µl) | %CD4 | Viral Load (copies/ml) | Time on HAART (months) | HAART regimen |
| --- | --- | --- | --- | --- | --- | --- |
| 1 | 336 | 250 | 20,85 | <20 | 150 | TDF/FTC+NVP |
| 2 | 204 | 440 | 40,3 | <20 | 96 | ABC/3TC+NVP |
| 3 | 33 | 930 | 30,9 | <20 | 9 | TDF/FTC+ATV/r |
| 4 | 41 | 1390 | 40,6 | <20 | 19 | ABC/3TC+RAL |
| 5 | 19 | 1310 | 42,30 | <20 | 12 | TDF/FTC/EFV |
| 6 | 62 | 650 | 18,3 | <20 | 8 | TDF/FTC+EVG/c |
| 7 | 44 | 790 | 35,4 | <20 | 12 | TDF/FTC+EVG/c |
| 8 | 51 | 1120 | 40,2 | <20 | 28 | TDF/FTC+DRV/r |
| 9 | 19 | 360 | 21,09 | <20 | 1 | TDF/FTC+EVG/c |
| 10 | 31 | 320 | 20,81 | <20 | 23 | ABC/3TC+DTG |
| 11 | 26 | 1450 | 54,99 | <20 | 19 | TDF/FTC+RPV |
| 12 | 42 | 730 | 40,32 | <20 | 21 | TDF/FTC+RPV |
| 13 | 28 | 1300 | 50,73 | <20 | 19 | ABC/3TC+DTG |
| 14 | 9 | 630 | 34,84 | <20 | 8 | TDF/FTC+EVG/c |
| 15 | 18 | 880 | 41,35 | <20 | 6 | ABC/3TC+DTG |
| 16 | 30 | 940 | 47,07 | <20 | 20 | TDF/FTC+EVG/c |
| 17 | 31 | 840 | 35,75 | <20 | 23 | TDF/FTC+EVG/c |
| 18 | 18 | 1520 | 46,83 | <20 | 6 | ABC/3TC+ATV/r |
| 19 | 47 | 800 | 40,42 | <20 | 23 | ABC/3TC/RAL |
| 20 | 48 | 850 | 40,64 | <20 | 27 | TDF/FTC+RPV |
| 21 | 25 | 1030 | 34,87 | <20 | 16 | TDF/FTC+EVG/c |
| 22 | 73 | 1410 | 36,46 | <20 | 24 | TDF/FTC+EVG/c |
| 23 | 48 | 490 | 36,57 | <20 | 18 | ABC/3TC+RPV |
| 24 | 280 | 330 | 15,98 | 78 | - | UNT |
| 25 | 71 | 850 | 25,8 | 5.950 | - | UNT |
| 26 | 68 | 480 | 26,4 | 77.700 | - | UNT |
| 27 | 22 | 550 | 26,4 | 8.600 | - | UNT |
| 28 | 84 | 240 | 6,51 | 1.820.000 | - | UNT |
| 29 | 335 | 290 | 37,4 | 94.100 | - | UNT |
| 30 | 198 | 170 | 5,23 | 517.000 | - | UNT |
| 31 | 1 | 80 | 9,89 | 148.000 | - | UNT |
| 32 | 2 | 450 | 20,1 | 1.020.000 | - | UNT |
| 33 | 96 | 780 | 51,9 | 2.050 | - | UNT |
| 34 | 213 | 350 | 22,7 | 74.900 | - | UNT |
| 35 | 2 | 180 | 4,19 | 417.000 | - | UNT |
| 36 | 62 | 830 | 43,7 | 51.900 | - | UNT |
| 37 | 2 | 850 | 35,17 | 559 | - | UNT |
| 38 | 270 | 280 | 17,5 | 189 | - | UNT |
| 39 | 75 | 690 | 33,1 | 4.220 | - | UNT |
| 40 | 315 | 150 | 11,51 | 49.300 | - | UNT |
| 41 | 0,1 | 150 | 7,5 | 5.000.000 | - | UNT |

FTC, emtricitabine; TDF, tenofovir; NVP, nevirapine; ATV/r, atazanavir boosted with ritonavir; 3TC, lamivudine; EFV, efavirenz; ABC, abacavir; RAL, raltegravir; EVG/c, elvitegravir boosted with cobicistat; DTG, dolutegravir; DRV/r, darunavir boosted with ritonavir; RPV, Rilpivirine; UNT, untreated.
